# Antibiotic Therapy Using Phage Depolymerases: Robustness Across a Range of Conditions

**DOI:** 10.3390/v10110622

**Published:** 2018-11-12

**Authors:** Han Lin, Matthew L. Paff, Ian J. Molineux, James J. Bull

**Affiliations:** 1 Department of Integrative Biology, University of Texas, Austin, TX 78712, USA; hanl@austin.utexas.edu (H.L.); matthew.paff@utexas.edu (M.L.P.); 2 Institute for Cellular and Molecular Biology, University of Texas, Austin, TX 78712, USA; 3 Department of Molecular Biosciences, University of Texas, Austin, TX 78712, USA; 4 LaMontagne Center for Infectious Disease, University of Texas, Austin, TX 78712, USA; 5 Center for Computational Biology and Bioinformatics, University of Texas, Austin, TX 78712, USA

**Keywords:** phage therapy, bacterial infection, capsule depolymerase, antibiotic, animal model, bacterial resistance

## Abstract

Phage-derived depolymerases directed against bacterial capsules are showing therapeutic promise in various animal models of infection. However, individual animal model studies are often constrained by use of highly specific protocols, such that results may not generalize to even slight modifications. Here we explore the robustness of depolymerase therapies shown to succeed in a previous study of mice. Treatment success rates were reduced by treatment delay, more so for some enzymes than others: K1- and K5 capsule-degrading enzymes retained partial efficacy on delay, while K30 depolymerase did not. Phage were superior to enzymes under delayed treatment only for K1. Route of administration (intramuscular versus intraperitoneal) mattered for success of K1E, possibly for K1F, not for K1H depolymerase. Significantly, K1 capsule-degrading enzymes proved highly successful when using immune-suppressed, leukopenic mice, even with delayed treatment. Evolution of bacteria resistant to K1-degrading enzymes did not thwart therapeutic success in leukopenic mice, likely because resistant bacteria were avirulent. In combination with previous studies these results continue to support the efficacy of depolymerases as antibacterial agents in vivo, but system-specific details are becoming evident.

## 1. Introduction

In the increasingly urgent search for new treatments against bacterial infections, phages and phage products hold promise [1,2,3]. Offsetting the now many laboratory studies of phages and phage products showing positive results [4,5,6], the few clinical phage therapy trials conducted under standards of Western medicine have actually failed [7,8,9]. The most recent randomized clinical phage therapy trial was also stopped prematurely for lack of efficacy, although small effects were deemed encouraging [10]. The contrasting outcomes between actual trials and laboratory infections raise the possibility that experimental studies poorly represent applications. One obvious concern in generalizing from experimental infections to clinical settings is the potential sensitivity of results to specifics of the experimental protocol. A step toward generality of a therapeutic agent is thus to broaden the experimental protocol and measure the sensitivity of treatment success to experimental variables. Here we evaluate the robustness of phage depolymerase therapies previously demonstrated to rescue mice from experimental infections of capsulated *E. coli* [11].

Several classes of phage proteins have exhibited antibiotic potency, including endolysins [12], viron-associated lysins [13], holins [5], spanins [14] and bacterial polysaccharide depolymerases [15]. Some endolysins are already commercially available and are currently being tested in clinical trials [3,16]. Capsular depolymerases represent an interesting type of antibiotic: they do not kill per se, but merely strip the bacteria of protective polysaccharides and thus expose the bacteria to immune components [17]. They have a potential advantage over endolysins in that they do not lyse the bacteria, thereby minimizing inflammatory responses from endotoxins [18]. In vivo studies of capsule depolymerases are yet limited but appear to generalize across different animal models (Table 1). Furthermore, enzymes that disrupt biofilms, in part by degradation of polysaccharide, but do not kill bacteria are also showing promise [19,20].

Our purpose here is to explore the robustness of capsular depolymerase enzymes in treating experimentally infected mice. The bacteria are *E. coli* with K1, K5 or K30 capsules, and the enzymes were obtained as purified proteins expressed from clones of phage genes. Previous work demonstrated therapeutic success of several enzymes when using (i) simultaneous infection and treatment, (ii) intramuscular administration, and (iii) immunocompetent mice [11]. Here we explore treatment efficacy when relaxing these conditions. By measuring performance of depolymerase treatments under different conditions and with different enzymes, our work also exposes possible realms for improving therapeutic efficacy of depolymerases, as in structure/function properties of the enzymes.

## 2. Materials and Methods

### 2.1. Bacterial Strains and Cell Culture

The pathogenic bacterial strains used in this study were K1-capsulated *E. coli* RS218 [28], K5-capsulated ATCC 23506, and K30-capsulated E69 [29]. *E. coli* lab strains used only for phage propagation or cloning were the K1-capsulated K12 strain EV36 [30] and BL21(DE3). Cells were generally grown in LB broth (10 g tryptone, 5 g yeast extract, 10 g NaCl per liter) in 37 °C shakers. Cell density was determined by plating serial cell dilutions on LB agar (1.3% *w*/*v*) plates for colony counts.

Capsule-free isolates of RS218 were selected by culturing RS218 on LB plates containing K1H phage [31]. Colonies that grew on the plates were picked, diluted in LB medium and replated on LB plates containing K1 phage. The colonies grown on these second plates were picked and cultured in LB medium containing K1 phage before mouse injection. The addition of phage was a precaution against any possible revertants to a capsulated state.

### 2.2. Coliphage Strains and Culture

Phages K1E, K1F and K1H [32] were propagated on host *E. coli* EV36. K1–5 [33] and K30 [34] were grown with host *E. coli* ATCC 23506 and E69, respectively. Phages were purified by equilibrium CsCl-gradient centrifugation and dialyzed into SM buffer (50 mM Tris–HCl, 100 mM NaCl, 8 mM MgSO_4_, pH 7.5) as previously described [11,35]. Phage titrations were performed by plaque counts on an appropriate host in LB soft agar (0.65%) overlay.

### 2.3. Plasmids, Protein Expression and Purification

The phage capsule depolymerases were expressed in *E. coli* BL21(DE3) with pET28b constructs containing cloned depolymerase genes as previously described [11]. Proteins were purified with HisPur Ni-NTA resin (Thermo Fisher Scientific, Rockford, IL, USA), and dialyzed into PBS buffer (137 mM NaCl, 2.7 mM KCl, 10 mM Na_2_HPO_4_, 1.8 mM KH_2_PO_4_, pH 7.5) with 3.5 kDa MWCO dialysis membranes (Spectrum-Repligen, Houston, TX, USA). Protein concentrations were estimated by absorption at 280 nm with a Nanodrop ND-1000 (Thermo Fisher Scientific, Wilmington, DE, USA).

### 2.4. Mouse Infection Model

Animal work was performed under NIH guidelines and the University of Texas IACUC protocols (AUP-2015-00035, AUP-2018-00010). Female NIH Swiss outbred mice (Envigo, Somerset, NJ, USA) aged 4–6 weeks with 20–25 g weights were used here in all studies. All intramuscular (IM) inoculations of bacteria used the left thigh; all IM inoculations of enzyme or phage used the right thigh. Mouse survival was monitored twice daily for 5 days.

The following experiments were undertaken.

Delayed treatment of normal mice with enzyme or phage. Mice received an IM injection of 100 μL bacteria, dosed at either 1.2–3.4 × 10^8^ CFU of RS218, 3.1–6.1 × 10^8^ CFU of ATCC 23506, or 1.1–2.8 × 10^8^ CFU of E69 per mouse. Enzyme or phage inoculation in the contralateral thigh followed at 8 h, dosed at 20 μg enzyme in 100 μL PBS or 10^7^ pfu phage in 100 μL SM buffer. A dose of 10^7^ phage is 10-fold lower than used in the delayed treatment studies of [36], but much higher than doses used in studies with simultaneous treatment [37]. Given the rate of phage amplification in the host [37], 10^7^ was expected to be highly effective.

Different administration routes for immediate enzyme treatment. Mice received IM 1.2–2.9 × 10^8^ CFU of RS218 in 100 μL volume, and then 20 μg K1E, 2 μg K1F or 2 μg K1H in 100 μL volume either by contralateral IM or IP (intraperitoneal) inoculation.

The leukopenic mouse model. Mice were rendered leukopenic by IP injection of cyclophosphamide (CP) at 150 mg/kg body weight 4 days prior and then at 100 mg/kg body weight 1 day prior to infection. To determine an approximate lethal dose of bacteria, different mice were inoculated across a range of bacterial doses in 10-fold increments spanning 10^3^–10^7^ CFU (RS218) or 10^4^–10^8^ CFU (capsule-free RS218 derivative). Simultaneous enzyme (20 μg K1E, K1H) or phage (K1H 10^7^ pfu contralateral) treatment was tested in leukopenic mice infected by RS218 at the lethal dose of 2.2–5.1 × 10^4^ CFU. Delayed enzyme (20 μg K1F, K1H) treatment was also tested in leukopenic mice 8 h after infection.

Mouse survival was analyzed by Kaplan-Meier survival curves using SPSS software, where the cumulative survival probability was plotted over the time course of 5 days and statistically evaluated by log rank test [25,38,39].

### 2.5. Capsule Isolation and Degradation Assay

Capsules were isolated as previously described [11,40] for degradation assays monitored by gel electrophoresis and Alcian Blue staining. To compare activity of purified K1 enzyme to enzyme activity of intact phage, 10–20 μg K1 capsule was mixed with serial dilutions (0.25–8 μg) of K1E, K1F, K1H enzyme or CsCl-purified cognate phage at the calculated amount of enzyme equivalents at 37 °C for 1 h before gel electrophoresis. Enzyme equivalents of phages were calculated from the molecular weight of each enzyme [41,42] and the fact that these phages contain six depolymerase trimers and thus 18 enzyme molecules per phage particle [35]. 10^10^ phage would thus have ~3 × 10^−2^ μg enzyme (varying somewhat among different enzymes). This calculation assumes that phage stocks plate at a particle:plaque ratio of 1; a reduced efficiency of plating would underestimate the amount of enzyme in the phage sample.

To compare levels of K1 enzyme activity in mouse blood, 100 μL PBS containing 20 μg K1E, K1F or K1H enzyme or no enyzme (control) was injected to mice by IM or IP. Mice were euthanized at 1 h or 24 h after injection and blood was collected to prepare serum. 10–20 μg K1 capsule was incubated with 10 μL K1E serum or 0.3 μL K1F, K1H serum at 37 °C overnight before gel examination. The volumes of serum used in each reaction were determined in preliminary tests to achieve a dynamic range of degradation differentiable by visual inspection. Reactions with control serum were included as negative control, while reactions with serial dilutions of enzymes added immediately to the control serum were included as positive control.

### 2.6. Resistance Competition Assay (RCA)

The assay measures the in vivo effects of treatment on bacterial numbers. It works by inoculating mice with a mixture of mostly treatment-sensitive bacteria and a small number of treatment-resistant bacteria [36]. Mice are either treated or not (the latter being controls). Resistant bacteria increase in frequency (relative to controls) to the extent that treatment suppresses the population of sensitive bacteria. For this assay, RS218 was mixed with the K5-capsulated ATCC 23506 (ratio approximately 99:1). Six mice were inoculated in the left thigh with the normal bacteria dose (~2.9 × 10^8^ cells) and either treated simultaneously with contralateral IM of 20 μg K1H enzyme (3 mice) or not treated (3 controls). At 4 h post infection, mice were euthanized. The left thigh was removed, homogenized in 10 mL buffer, and the suspension plated at different dilutions on both LB plates and on LB plates saturated with 10^7^ pfu K1H phage. The LB plates support growth of both K1 and K5 bacteria, whereas plates with phage grow only the K5-capsulated strain, allowing a determination of K5 (ATCC 23506) frequency. Calculations of the RCA value used the formula in [36]: RCA = ln [(p_t_ (1 − p_0_))/(p_0_(1 − p_t_))]/t, with p_0_ as the proportion of resistant bacteria in control mice and p_t_ as the proportion resistant bacteria in treated mice, both measured at time t (hours of treatment). For an RCA value of R, e^−R^ is the per hour growth of bacteria under treatment relative to growth without treatment.

## 3. Results

### 3.1. Delayed Treatment Reduces Efficacy

We reported that phage capsule depolymerases were broadly effective in treating lethal *E. coli* infections in mice [11]. That work applied treatment simultaneously with infection. Here we explored the efficacy of delayed treatment, as a delay might better represent clinical therapeutics.

**Figure 1:**
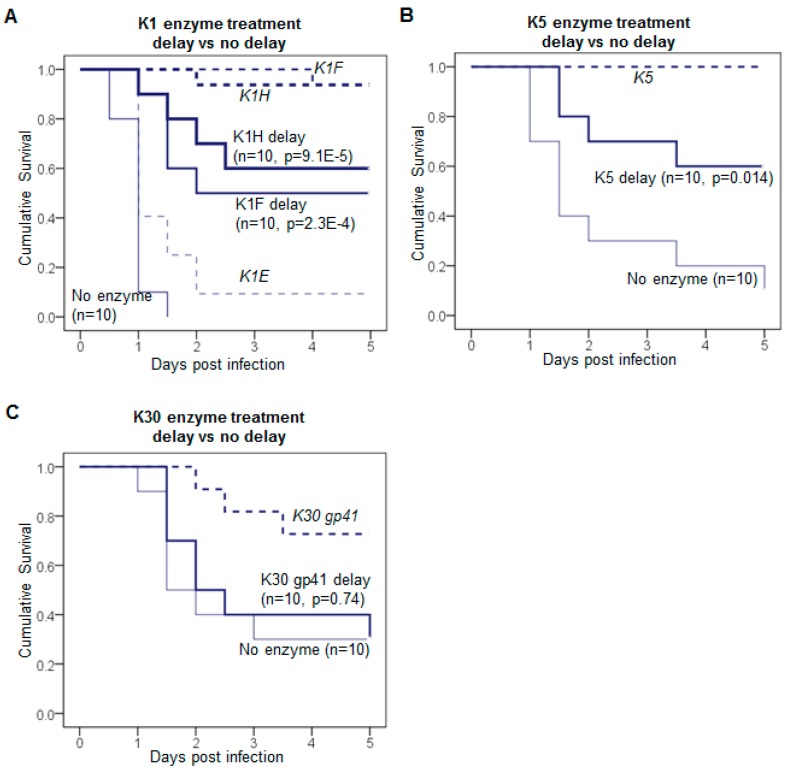
Delayed treatment of infection using capsule depolymerases. (**A**) 1.2–3.4 × 10^8^ CFU of K1-capsulated *E. coli* treated with 20 μg of K1F or K1H enzyme; (**B**) 3.1–6.1 × 10^8^ CFU of K5-capsulated *E. coli* treated with 20 μg of K5 enzyme; (**C**) 1.1–2.8 × 10^8^ CFU of *E. coli* E69 treated with 20 μg of K30 enzyme. All inoculations were IM (intramuscular, thigh); enzyme was administered 8 h after bacteria in the contralateral thigh. Mouse survival was monitored for 5 days and Kaplan-Meier survival curves in solid lines were plotted with the cumulative probability of survival over time for each treatment. Previously reported survival curves of simultaneous enzyme treatments are included for comparison (dashed lines). The mouse number (*n*) of each treatment is given for each curve. Log rank test: *p* values are listed for delayed treatments compared to the no-enzyme control.

Delayed treatment is better than no treatment only for some enzymes. The delayed treatments of phage capsule depolymerases given 8 h after infection exhibited reduced efficacy (Figure 1). The delayed treatment with K1F or K1H enzyme resulted in 50–60% survival compared to zero survival in control (Figure 1A), while the simultaneous treatment led to 90–100% survival [11]. K1E did not rescue well in simultaneous treatment, thus the delayed treatment was not tested here. Delayed K5 enzyme treatment showed a 60% survival (Figure 1B) compared to 100% survival when treatment was immediate; both were significantly better than no-treatment controls. Delayed treatment with the least active enzyme K30 gp41 did not significantly improve survival (Figure 1C).

**Figure 2:**
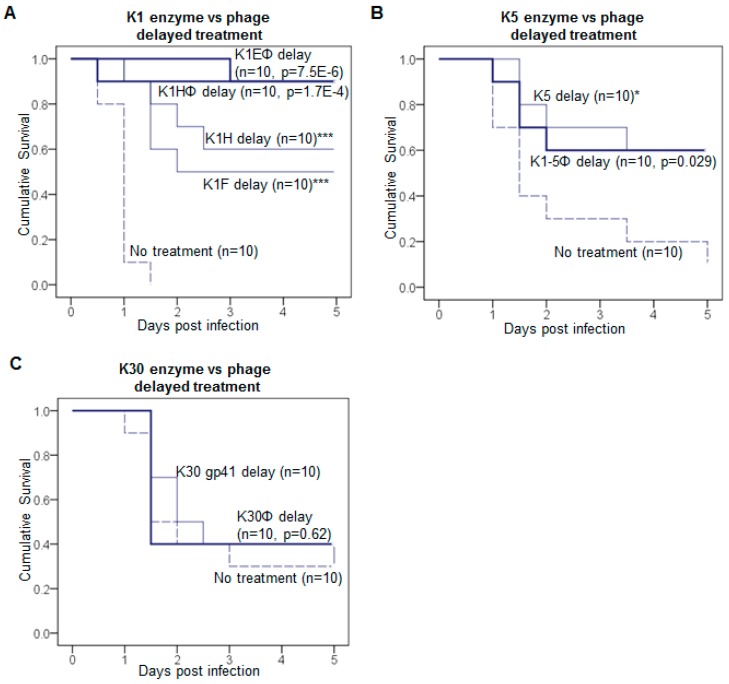
Comparison of depolymerases and cognate phages in treatment. Delayed treatments using phages were carried out in parallel to depolymerase. (**A**) K1E and K1H phage; (**B**) K1-5 phage; (**C**) K30 phage. Depolymerase data are from Figure 1. For phage treatment, mice were infected with intramuscular (IM) inoculations; 8 h later they received ~10^7^ pfu phage IM in the contralateral thigh. For comparison to depolymerase treatment, this dose of phage carries about 3 × 10^−5^ μg of depolymerase (see Methods for calculation). Kaplan-Meier survival curves were plotted as in Figure 1. The mouse number *n* of each treatment is labeled on each curve. Log rank test: *p* values for delayed phage treatments compared to the no treatment control are listed, or * *p* < 0.05, *** *p* < 0.001 for delayed enzyme treatment as in Figure 1.

Phages outperform enzyme only for K1 bacteria. Phages might be expected to outperform enzymes on the grounds that phage have multiple effects: They amplify within the host, they kill, and cell lysis releases free enzyme as unassembled tailspikes. Yet a superiority of phages under delayed treatment was observed only for K1-capsulated bacteria. K1H or K1E phage yielded higher survival rates than K1 enzymes in delayed treatment (Figure 2A). In contrast, K5 or K30 phages were no better than their enzymes in delayed treatment (Figure 2B,C). As a control for the effects of phage amplification, K1F phage, which does not propagate on RS218, had no effect in mice (data not shown), whereas K1F enzyme efficacy was similar to that of K1H (Figure 1A).

**Figure 3:**
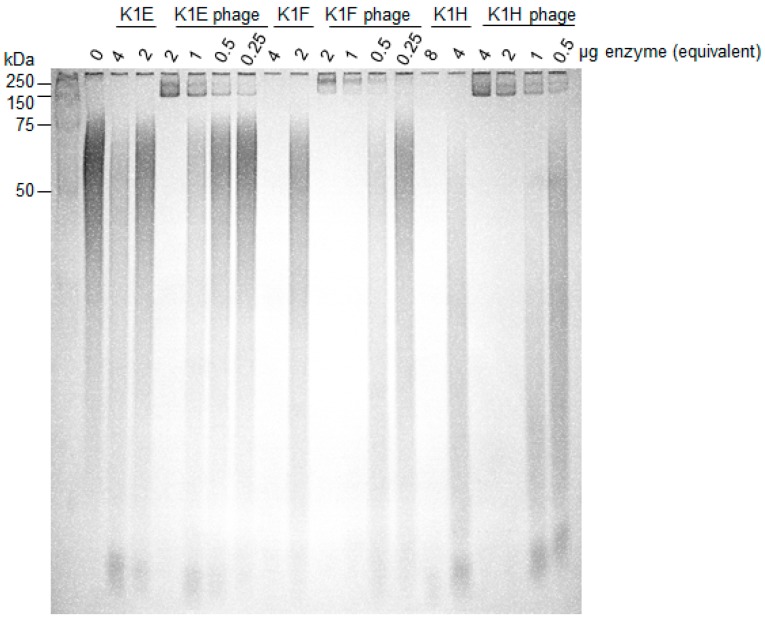
In vitro activity comparison of depolymerases and cognate phages. 10–20 μg of K1 capsule were incubated with serial dilutions of K1E, K1F, K1H depolymerase or phages at the indicated amount of enzyme equivalents (0.25–8 μg). Active enzyme is indicated by loss of signal within the lane; enzyme associated with phages is 4–8 fold more active than free enzyme. Incubation was at 37 °C for 1 h; reactions were fractionated using 12% TBE-PAGE with Alcian Blue staining. Protein standards were loaded in the leftmost lane and their molecular weights indicated.

Phages carry active depolymerases as tailspikes that are used in adsorption and penetration of the capsule. It is possible that the activities of the purified enzymes were substantially less than the activities of tailspikes on intact phages. We thus compared the in vitro degrading activities of purified enzyme with activities of intact phages (Figure 3). Degradation by phages was 4–8 fold better than degradation by molar equivalents of purified enzyme. Perhaps surprisingly, in view of the different in vivo performances of the enzymes, in vitro differences among the three K1-specific phages were small.

### 3.2. Efficacy Varies with Route of Administration and Enzyme

K1E enzyme rescued rat pups by IP inoculation [21,22] but was minimally effective in mice by IM inoculation [11]. It does not appear that our purified K1E enzyme is at fault—its in vitro activity approximately matches that of the other K1 enzymes (Figure 3). Motivated by suggestions that administration routes may affect drug bioavailability [43,44,45], we compared the efficacy of IM versus IP administration routes of the different K1 enzymes; these studies used immediate treatment.

**Figure 4:**
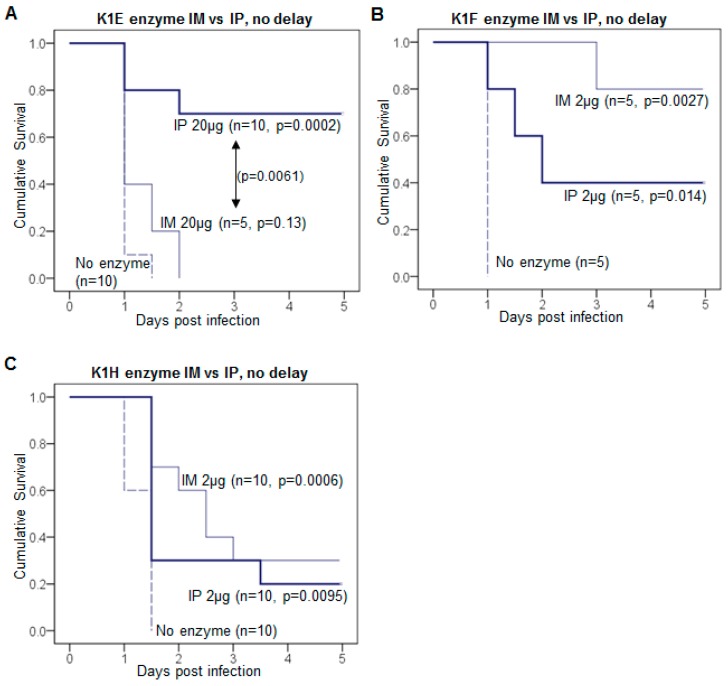
Administration route can affect treatment efficacy. (**A**–**C**) Kaplan-Meier survival curves of depolymerase treatment comparing intramuscular (IM) to intraperitoneal (IP) inoculation in mice. (**A**) Treatment with 20 μg K1E; (**B**) Treatment with 2 μg K1F; (**C**) Treatment with 2 μg K1H. In all mice, 1.2–2.9 × 10^8^ CFU of K1-capsulated *E. coli* were injected IM, followed by inoculation of enzyme either IM in the contralateral thigh or IP. Mouse survival was monitored for 5 days and the cumulative probability of survival was plotted for each treatment. The mouse number *n* of each treatment is labeled by each curve. Log rank test: *p* values between enzyme treatment and the no-enzyme control, or significant *p* values between IM and IP treatment are listed.

Different enzyme efficacies in mice by different administration routes. Following an IM inoculation of RS218 bacteria, K1E depolymerase efficacy was significantly higher for IP than for IM inoculation at the high dose of 20 μg (Figure 4A). Using only 2 μg for the more active (in vivo) K1F enzyme (Figure 4B) the opposite pattern is suggested, but the small sample sizes provide little power in significance tests. K1H (2 μg dose) yielded similarly low rescue rates for both routes of administration (Figure 4C).

**Figure 5:**
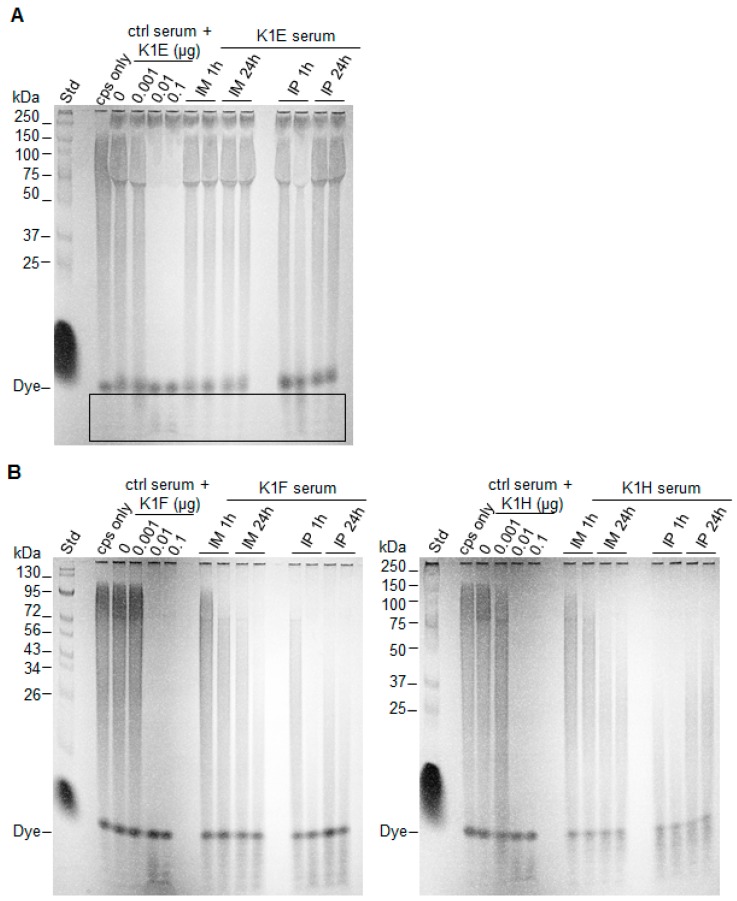
Comparison of depolymerase serum activity using different administration routes. Except for the molecular weight standard (“std”) on the left of each panel, each lane fractionates 10–20 ug of K1 capsule (“cps only”) or an overnight reaction of capsule incubated with serum and/or enzyme. Lanes with “serum” used serum from mice inoculated with enzyme (**A**) K1E; (**B**) K1F (left) or K1H (right) (IM or IP, 1 mg/kg weight) and sacrificed at 1 or 24 h. Two mice were tested for each route and time point, shown in separate lanes. Lanes with “ctrl serum” had different amounts of free enzyme added to the control serum (from mice not receiving enzyme injection), as the control reactions. Only a slight activity of K1E is evident, and then only for IP 1h (bottom of lane, boxed). In contrast, K1F and K1H both exhibit clear activity by both routes of administration, with a possible effect of route for K1H at 1 h.

Basis of the effect of administration route. We tested capsule degradation activity of serum from mice that had been inoculated with enzyme by different routes. If bioavailability of enzyme is affected by route of administration, then serum from mice inoculated by the IP route should have different activity per unit volume than serum from mice inoculated IM. Consistent with treatment efficacy differences, detectable degradation by K1E serum was observed only for the IP route, and then only at 1 h post inoculation (Figure 5A, boxed region). As for K1F and K1H sera, IP delivery resulted in slightly higher capsule degradation activity than IM at 1 h post inoculation, and activity was largely maintained after 24 h exposure (Figure 5B,C). However, both K1F and K1H sera exhibited much higher activities than K1E serum, independent of administration route.

### 3.3. K1: Treatment Is Successful with Leukopenia

The above models used immunocompetent mice, requiring high inocula of bacteria to overwhelm innate immunity. To better represent infections that progress from low to high concentrations of bacteria, we tested mice that had been rendered leukopenic, limiting the studies to K1-capsulated bacteria.

**Figure 6:**
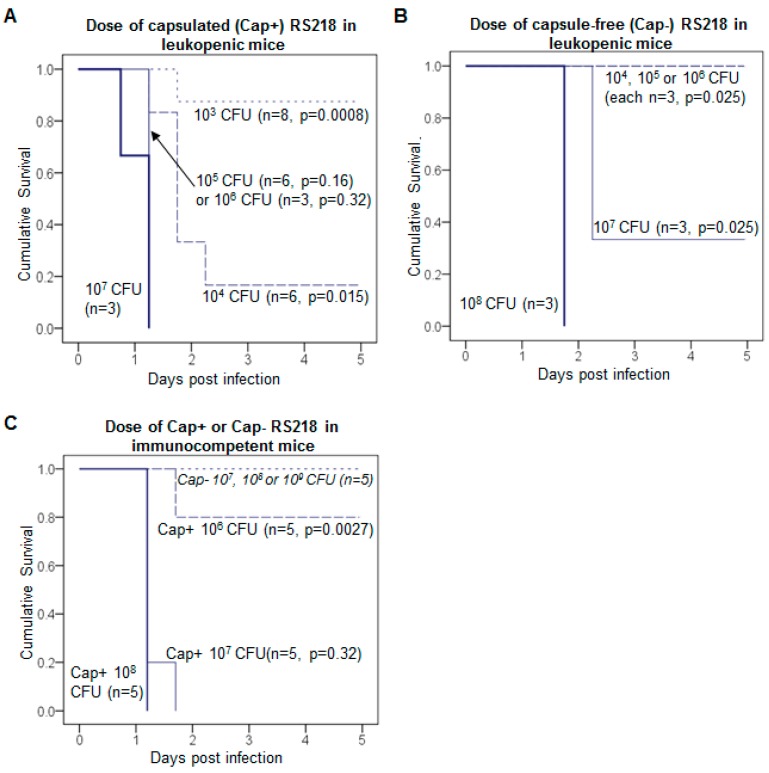
Capsule-free RS218 is avirulent in mice. Kaplan-Meier survival curves of mice per dose of different strains. (**A**) K1-capsulated RS218 (Cap+) in leukopenic mice; (**B**) capsule-free RS218 (Cap−) in leukopenic mice; (**C**) Cap+ and Cap− RS218 in immune competent mice. The median lethal dose is 10^3^–10^4^ CFU for Cap+ and 10^6^–10^7^ CFU for Cap- RS218 in leukopenic mice, a difference of 3 orders of magnitude. The median lethal dose in normal mice is 10^6^–10^7^ CFU for Cap+ RS218, while Cap− RS218 is not lethal at doses as high as 10^9^ CFU. Mouse survival was monitored for 5 days and the cumulative probability of survival was plotted for each dose. The mouse number *n* of each treatment is labeled by each curve. Log rank test: *p* values are listed for lower doses compared to the highest dose of each strain.

**Figure 7:**
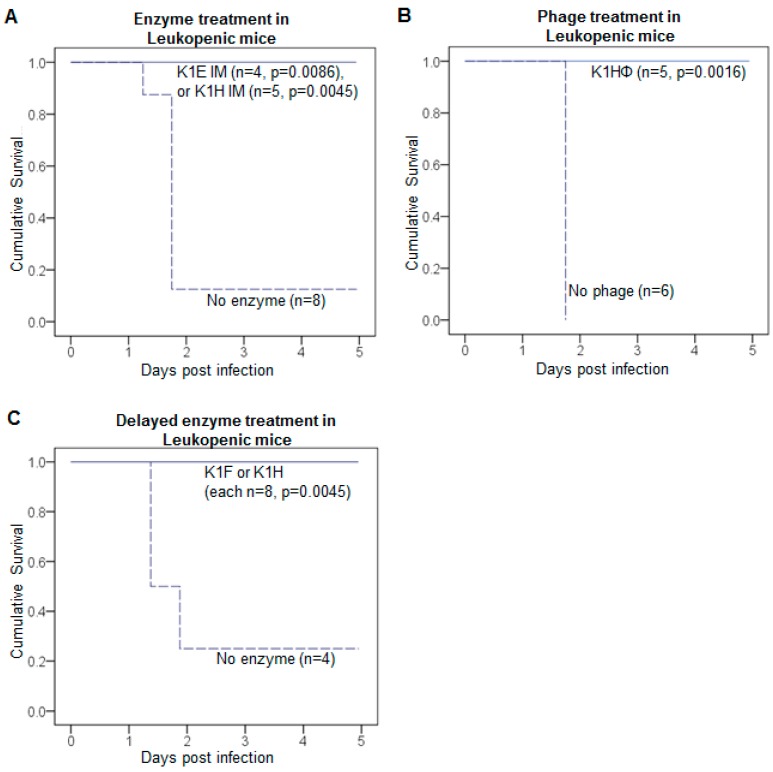
Enzymes and phages rescue infections of leukopenic mice. Total rescue of infections by K1 *E. coli* in leukopenic mice was achieved by simultaneous treatment with (**A**) K1E or K1H depolymerase; (**B**) K1H phage, and by delayed treatment with (**C**) K1F or K1H depolymerase. Each panel shows Kaplan-Meier survival curves with sample sizes given by *n*. Infection was initiated by intramuscular (IM) inoculations of 2.2–5.1 × 10^4^ CFU of K1 *E. coli* in the left thigh. Treatment was an IM injection of 20 μg enzyme or 10^7^ phage in the contralateral thigh. Treatment was administered either (**A**,**B**) immediately at the time of infection or (**C**) 8 h after infection. Mouse survival was monitored for 5 days and the cumulative probability of survival was plotted for each treatment. Log rank test: *p* values are listed for each treatment compared to no-treatment control.

Leukopenic mice are far more susceptible to RS218 than are immune-competent mice (Figure 6). A bacterial inoculum slightly exceeding 10^4^ was fatal in the leukopenic mice (Figure 6A), compared to the lethal threshold dose of 10^6^–10^7^ in immunocompetent mice (Figure 6C). In leukopenic mice, doses above 10^4^ cfu reduced survival time, to about a half day shorter using 10^7^ bacteria (Figure 6A). Phage and enzyme each rescued the leukopenic mice in most cases, whether with immediate (Figure 7A,B) or delayed treatment (Figure 7C).

Avirulence of capsule-free bacteria. Our use of leukopenic mice was motivated to assess the potential for treatment failure via the evolution of treatment-resistant bacteria, as observed in a neutropenic mouse-*Pseudomonas* infection model [39]. Resistance is a potential cause of treatment failure with both phages and antibiotics [39,46,47,48,49,50]. Treatment success in our studies indicates that resistance did not ascend, at least not enough to affect survival (true both for immunocompetent and leukopenice mice). We thus addressed the reason evolution of resistance was not a problem in our system despite its cause of treatment failure in others. In the case of enzyme treatment, the relevant “resistance” phenotype is presumably absence of a capsule, since the enzyme has no substrate on those cells; that phenotype is also resistant to phages encoding K1 enzymes [51]. Therefore, the lethal consequences of a capsule-free RS218 derivative in leukopenic mice was evaluated over a series of inoculum sizes (Figure 6B,C). The capsule-free bacteria are profoundly less virulent: The mice could survive an inoculum 10^3^–10^4^ times larger of capsule-free bacteria than of capsulated bacteria. Thus, resistant (capsule-free) bacteria are more easily controlled by the immune system than are treatment-sensitive, capsulated bacteria and thus are not a reason for treatment failure. (The same argument applies to cells resistant to treatment with phages that require the capsule.) Smith and Huggins (1982) observed reduced virulence of K1 capsule-free bacteria in immunocomptent mice, so these results generalize across both immunocompetent and immune-compromised mice.

Successful, delayed treatment of leukopenic mice. For immunocompetent mice, immediate treatment was superior to delayed treatment for all enzymes. For K1-capsulated bacteria, delayed treatment was still better than the absence of treatment but not as good as immediate treatment. The inferiority of delayed treatment might stem from an artefact of the infection model, specifically that such a high dose of bacteria must be introduced so that the window of opportunity for treatment is short. From the work presented above of lethal inoculum doses in leukopenic vs immunocompetent mice (Figure 6C), leukopenia increases the dynamic range of bacterial densities in which to evaluate treament efficacy—a lower inoculum can be used and the time available to treat increases. To test this latter premise, we attempted delayed treatment of leukopenic mice using K1 enzymes. The bacterial inoculum was approximately 5 × 10^4^ CFU, and enzyme treatment (20 μg K1F or K1H) was given at 8 h. All 16 treated mice survived, whereas only 1 of the 4 controls survived (Figure 7C). This survival rate is significantly higher than that with delayed treatment of immunocompetent mice, but a direct comparison of delayed treatment between leukopenic and immunocompetent mice is not easily interpreted (see Discussion).

### 3.4. Measuring the Dynamical Impact of Treatment: Resistance Competition Assay

Survival is an endpoint measure of efficacy but gives little insight to the underlying process of bacterial dynamics. Levin [36] developed a metric of the quantitative impact of treatment on bacterial abundances—the resistance competiton assay (RCA). Mice are inoculated with a mix of two bacterial strains, one sensitive and the other resistant to treatment. In untreated controls, the two strains grow in vivo according to their intrinsic abilities, though not necessarily at the same rate. With treatment, the sensitive strain is specifically inhibited. Relative to controls, the resistant strain thus increases its proportion, and the magnitude of this increase depending on how much the sensitive strain is inhibited or killed. An RCA value greater than 0 indicates that the treatment suppresses the sensitive strain, higher values moreso.

Six mice were used for an RCA measure of K1H enzyme, 3 controls and 3 treatments. The resistant strain was the K5-capsulated strain, one that is virulent in vivo (as opposed to a capsule-free RS218). Initial frequency of the resistant strain in the mix was 0.006. At four hours, the frequencies of resistant cells were significantly higher in the enzyme-treated mice than in the controls (Table 2), and the RCA value was 0.30.

## 4. Discussion

This study broadly supports a growing realization that phage depolymerases might be useful therapeutics against particular kinds of bacterial infections. The work here also starts to put bounds on the degree of generality in depolymerase utility. Our study used 3 different capsulated *E. coli* strains and tested 3 enzymes against K1 capsules, one enzyme against K5, and one enzyme against K30. The work presented extends tests of therapeutic efficiency to varying conditions and may thus provide insight to efficacy in clinical infections.

K1 depolymerases that performed well in immunocompetent mice also performed well in immune-compromised, leukopenic mice. As expected, leukopenic mice were far less tolerant of bacteria than were immunocompetent mice. Given the artificiality of mouse infections with high doses of bacteria, leukopenic mice may yield a more realistic infection model than do immunocompetent mice, chiefly by increasing the dynamic range of bacterial densities that overwhelm the immune response. In support of this interpretation, leukopenic mice exhibited a modest increase in survival time (when inoculated with low numbers of bacteria), intramuscular treatment with K1E enzyme was improved with leukopenia (Figure 7A), and leukopenic mice were more easily rescued with delayed treatment than were immunocompetent mice. We caution, however, that differences in treatment efficacy between immunocompetent and leukopenic mice are not easily interpreted because of the many differences in the two model infections. Even so, the greater range of useful inoculum sizes afforded by leukopenia increases the latitude of experimental designs.

In contrast to observations with *Pseudomonas* infections of neutropenic mice treated with phages [39], evolution of resistance to K1 capsular depolymerases did not thwart treatment success in leukopenic mice. The absence of resistance evolution likely stems from the avirulence of capsule-free bacteria. The bacterium’s only reasonably accessible evolutionary path to avoid the depolymerase is to lose the capsule (it cannot easily generate a new capsule type). This would leave the resistant cell in the same phenotypic state as an otherwise capsulated cell that was stripped of its capsule by enzyme. Thus resistant bacteria and enzyme-treated bacteria may be functionally equivalent in the mouse.

On the negative side for enzyme therapy, delayed treatment of immune competent mice not only reduced efficacy but the reduction was complete for the K30 depolymerase, which lost all efficacy under delayed treatment. However, K30 was also the enzyme showing the weakest effect under simultaneous treatment (e.g., it required the highest dose of all enzymes to rescue mice; Lin et al., 2017 [11]) so there was a strong *a priori* basis for anticipating the large effect of delay with K30 enzyme. In contrast, delayed administration of two K1 enzymes retained efficacy, albeit at a reduced level. Perhaps significantly, with delayed treatment, the two cognate K1-specific phages achieved higher rescue than enzymes. Although no negative consequence of delayed treatment was observed in leukopenic mice (tested only for K1 depolymerases), the outcome with immune competent mice likely reflects a true negative effect of delayed treatment that would be manifested in other contexts.

To gain additional insight to enzyme efficacy, we conducted a resistance competition assay (RCA). RCA values of 2.1 and 1.7 were reported for immediate treatment with streptomycin and a phage requiring the K1-capsule, both highly effective [36], whereas values ranging from 0.2–0.5 were reported for treatments that were only somewhat effective. The observed RCA value for immediate K1H depolymerase treatment determined here is 0.3; over the course of 10 h, this translates into a 95% reduction of bacterial numbers relative to the no treatment control.

The RCA value for K1H, an enzyme that is highly effective in rescuing mice, is thus somewhat lower than expected from previous results. We can suggest several possible reasons for this discrepancy. First, immediate treatment with enzyme may not be quite as effective as with streptomycin or the K1-dependent phage—all treatments yield near 100% survival, but differences could be masked at this upper limit of the dynamic range; indeed, the enzymes do not invariably rescue [11]. Second, treatment spans a time course, and the RCA is measured at a point in time, so 4 h may not be the best time for comparison (e.g., enzyme may be slower to diffuse than antibiotic). Third, assays with phage treatment risk inflating the RCA by allowing bacterial killing to continue during the thigh-processing step. Finally, in comparison to phages, enzymes do not lyse cells and release toxins, so recovery with enzyme treatment may be feasible with a lower dose of killing than is required with phages. Although we cannot yet suggest whether any of these explanations has merit, the comparisons provide interesting avenues for further study.

A greater therapeutic efficacy of phages than of cognate depolymerase enzyme is not surprising, given that phages amplify on the bacteria, whereas enzyme concentration remains static or, more likely, declines due to inactivation and clearance. However, understanding the basis of greater phage efficacy is non-trivial because phage amplification has several components relevant to treatment—direct bacterial killing from phage infection, release of progeny phage particles that contain fresh enzyme molecules, plus the release of additional free enzyme in the form of unassembled tailspikes at the time of cell lysis. A phage inoculum of 10^7^ pfu (used here) carries about 2.5–3 × 10^−5^ μg enzyme, hence ~1.3 × 10^−6^ less depolymerase delivered in the phage inoculum than delivered in our enzyme treatment. Even with this profound difference and only 4–8 fold greater activity of phage virion-associated enzyme than of the purified form, K1H or K1E phage still gave rise to better survival rates than purified K1 enzymes. This greater efficacy of phages could speak either to a benefit of direct killing or to possibly orders of magnitude greater enzyme produced as the phage population expands. We of course don’t know the balance between these two effects. Yet, whatever argument is put forth for the superiority of K1 phages (except K1F, which does not grow on the K1-capsulated host used here) under delayed treatment, K5 phage or K30 phage did not improve survival rate over their pure enzymes at the doses tested.

Reduced efficacy with delayed treatment of any form is not surprising, especially with infections that are rapidly lethal. However, although it is tempting to interpret the effect of a treatment delay as merely giving the bacteria a “head start” to reach a lethal threshold, previous work using K1 infections of mice showed that the bacteria changed state with time to become recalcitrant to treatment [36]. This change in susceptibility may be due to the bacterial environment suddenly changing from laboratory growth media to mouse tissue, as altered bacterial physiology and community lifestyle could affect phage infection [52] and probably phage product treatment. Thus, the effect of therapeutic treatment delay could be one of the bacteria becoming more difficult to treat than of them being more numerous. The use of leukopenic mice as an experimental infection model may help avoid that complication and yield therapeutic regimes more redily translatable to clinical use.

A surprising result was the effect of route of administration on efficacy, at least for enzyme K1E. It is of course easy to argue that each enzyme has its own biochemical properties that may affect pharmacokinetics. For example, unlike the other K1 enzymes used here, K1E purified under our protocol tends to form 18-mers instead of trimers [11]. The larger enzyme complex might affect in vivo distribution and result in poor IM treatment efficiency, but this can only be argued on a *post facto* basis. Whatever the cause, the result indicates that treatment “failure” by one route does not imply failure by other routes.

By applying modifications of animal models, we have obtained more detailed understanding of how phage biology and enzyme biology complicate the pharmacokinetic and pharmacodynamic properties of phage and enzyme in infection treatment. The complex biological and pharmacological properties of phages is one major reason holding back whole phages from drug development and approval [53]. Phage derived enzymes are more similar to conventional antibiotics and thus more suitable than whole phages for the current drug approval processes even if not necessarily more suitable for treatment efficacy. In contrast to phages, depolymerases do not lyse bacteria and thus do not release endotoxin, so there may be circumstances in which enzymes are superior to phages [54,55]. A better understanding of enzyme structure and enzyme kinetics could greatly advance the development and approval of phage-derived enzymes as a novel class of antibiotics.

## Figures and Tables

**Table 1:** In vivo studies of capsule depolymerases in animal infection models.

Capsule Depolymerases	Animal Infection Model	References
EndoE (K1E)	*E. coli*; neonatal rats	Mushtaq et al., 2004 [21]; 2005 [22]
CapD; EnvD	*B. anthracis*; mice	Scorpio et al., 2008 [23]; Negus et al., 2015 [24]
K1-ORF34; K64dep	*K. pneumoniae*; mice	Lin et al., 2014 [25]; Pan et al., 2015 [26]
depoKP36	*K. pneumoniae*; moth larvae	Majkowska-Skrobek et al., 2016 [27]
K1F, K1H, K5, K30	*E. coli*; mice	Lin et al., 2017 [11]

**Table 2:** Frequencies of resistant bacteria in the Resistance Competition Assay.

Control Mice	Treated Mice ^1^	Average RCA
0.001, 0.0008, 0.0029	0.0052, 0.0049, 0.0047	0.30

^1^ A *t*-test comparing (logged) frequencies of treated mice versus control mice = 3.3 (4 df), *p* = 0.015 (1-tailed). A non-parametric combinations test of the perfect association of high frequencies in treated mice gives *p* = 0.05.
